# Human Metapneumovirus (HMPV): Advances in Diagnosis, Molecular Epidemiology, and Clinical Impact of an Underrecognized Respiratory Virus

**DOI:** 10.3390/diagnostics16101444

**Published:** 2026-05-09

**Authors:** Helal F. Hetta, Rehab Ahmed, Abdul Haseeb, Salwa Qasim Bukhari, Zinab Alatawi, Ahmad J. Mahrous, Mahmoud E. Elrggal, Ali M. Atoom, Yasmin N. Ramadan, Ahmed A. Kotb

**Affiliations:** 1 Division of Microbiology, Immunology and Biotechnology, Department of Natural Products and Alternative Medicine, Faculty of Pharmacy, University of Tabuk, Tabuk 71491, Saudi Arabia; rahmed@ut.edu.sa; 2 Department of Pharmacy Practice, Faculty of Pharmacy, University of Tabuk, Tabuk 71491, Saudi Arabia; ahanif@ut.edu.sa; 3 Department of Diagnostic Radiology, Faculty of Medicine, University of Tabuk, Tabuk 71491, Saudi Arabia; s.bukhari@ut.edu.sa; 4 Department of Family and Community Medicine, Faculty of Medicine, University of Tabuk, Tabuk 47512, Saudi Arabia; zalatawi@ut.edu.sa; 5 Department of Pharmaceutical Practices, College of Pharmacy, Umm Al-Qura University, Makkah 21955, Saudi Arabia; ajmahrous@uqu.edu.sa; 6 College of Medicine, Al Qunfudah Umm Al-Qura University, Al Qunfudhah 28821, Saudi Arabia; merggal@uqu.edu.sa; 7 Faculty of Allied Medical Sciences, Hourani Center for Applied Scientific Research, Al-Ahliyya Amman University, Amman 19111, Jordan; a.atoom@ammanu.edu.jo; 8 Department of Microbiology and Immunology, Faculty of Pharmacy, Assiut University, Assiut 71515, Egypt; yasmine_mohamed@pharm.aun.edu.eg (Y.N.R.); ahmedabedelaziz994@aun.edu.eg (A.A.K.)

**Keywords:** human metapneumovirus, HMPV, acute respiratory tract infection, pneumovirus

## Abstract

Human metapneumovirus or HMPV is an important respiratory pathogen of public health significance that primarily affects the immunocompromised, the very old, and young infants. However, recent studies have long since dispelled the idea that healthy adults are not at risk for serious sequelae, though it seems that HMPV has a particular affinity to infect children rather than adults. HMPV was first identified in 2001 and is implicated in a range of respiratory illnesses, from less severe upper respiratory infections to more severe pneumonia. This review compiles the recent literature on the epidemiology, molecular virology, and clinical characteristics of HMPV with an emphasis on, importantly, the virus’s significant contribution to respiratory morbidity and the requirement for better diagnostic capabilities and public health measures against this very much underappreciated viral pathogen.

## 1. Introduction

Human metapneumovirus or HMPV is a rising respiratory pathogen that has shown significant clinical and public health impact over the last few years. The virus has just been discovered in the past two decades. However, some serological investigations demonstrate that the virus has been circulating among humans for at least 70 years [1]. HMPV is similar to RSV (Orthopneumovirus), a member of the Pneumoviridae family. Although they are related, they have different epidemiological and clinical characteristics.

HMPV causes several respiratory conditions ranging from minor upper respiratory tract infections like the common cold to severe respiratory tract infections like bronchitis and pneumonia. Susceptible groups for this type of infection are immunocompromised individuals, the elderly, and infants, all of whom are more prone to exhibiting the symptoms of severe disease [2]. The virus caused severe pneumonia in immunocompetent elderly patients. This highlights the need for greater clinical awareness and consideration of this commonly neglected pathogen in differential diagnoses [3].

Although these groups are more susceptible to HMPV, there are reports of severe infection with the virus among previously healthy individuals of different ages, and due to that, there is a need to increase the awareness of HMPV infection in all populations [4,5].

Human metapneumovirus (HMPV) is the second most common cause of severe pneumonia in children under five, behind respiratory syncytial virus (RSV), according to the 2024 Pneumonia Etiology Research for Child Health (PERCH) project [6,7,8,9]. The frequent co-occurrence of HMPV with bacterial infections and its significant influence on pneumonia-related morbidity and mortality were highlighted in another investigation [6]. According to an investigation, 11% of hospitalized patients with HMPV required ventilatory assistance, and 12% needed ICU hospitalization [1]. In another investigation that examined more than 155,000 pediatric hospitalizations for acute respiratory tract infections (ARTI) over a 12-year period, researchers showed that severe HMPV cases were mostly found in infants younger than one year old and in children with comorbidities, with intensive care unit admissions accounting for 2.34% of pediatric cases with HMPV [10]. Most of the studies related to severe HMPV infection and its related disorders have been conducted on young infants, which has led to a lack of comprehensive data about HMPV infection and its clinical impact in adult cases.

Another study challenges the widely held notion among health care practitioners that the only people at risk are those who are immunocompromised or have significant comorbidities. A four-year prospective investigation that included about 1400 hospitalized patients revealed that HMPV caused 8% of acute respiratory illnesses and their hospitalizations, which is higher than other respiratory viruses, such as influenza A, and is comparable to RSV, the other member of its family [3,11]. This study highlighted the significant morbidity related to this virus, as it showed hospital stays of an average of nine days, ICU admission rates of 13.2 percent, and mortality rates slightly lower than those of RSV and influenza [3,11].

Despite the growing body of literature on human metapneumovirus (HMPV), several critical gaps remain in the current understanding of its clinical and public health significance. Recent studies have highlighted evolving epidemiological patterns, including shifts in genotype predominance, changes in seasonal dynamics following the COVID-19 pandemic, and increasing recognition of HMPV-associated morbidity in adult and immunocompetent populations. However, existing reviews often focus on isolated aspects such as virology or pediatric disease, with limited integration of molecular epidemiology, clinical impact across age groups, and emerging diagnostic and therapeutic approaches. Furthermore, uncertainties persist regarding viral transmissibility, genotype–severity relationships, and the development of effective vaccines. Therefore, a comprehensive and updated synthesis of recent advances is needed to bridge these gaps and provide a more cohesive understanding of HMPV as an underrecognized but clinically significant respiratory pathogen.

## 2. Literature Search Strategy

A comprehensive literature search was conducted to identify relevant studies on human metapneumovirus (HMPV). Electronic databases, including PubMed, Scopus, and Web of Science, were searched for articles published up to January 2026. The search strategy incorporated combinations of keywords such as “human metapneumovirus,” “HMPV,” “epidemiology,” “clinical characteristics,” “molecular epidemiology,” “diagnosis,” and “treatment.”

Studies were included if they were published in English and provided relevant data on HMPV epidemiology, molecular characteristics, clinical manifestations, diagnostic approaches, or therapeutic strategies. Both original research articles and review papers were considered. Priority was given to recent studies (published within the last 5–10 years), while older landmark studies were included where necessary to provide foundational context.

Articles were excluded if they lacked sufficient methodological detail, were not peer-reviewed, or were not directly relevant to the scope of this review. The selection of studies was based on their scientific relevance and contribution to the understanding of HMPV.

## 3. Molecular Characteristics

HMPV, a pleomorphic virion with a size ranging from 150 to 600 nm, has a similar genetic makeup to other members in the Paramyxoviridae family [12,13]. Its genetic structure is very similar to avian pneumovirus (aMPV), especially type C. Also, its genome has significant similarities with the human respiratory syncytial virus, with small differences in the gene arrangement and absence of non-structural genes in HMPV’s genome.

Both nonstructural proteins (NS1, NS2), strong multifunctional antagonists of the interferon (IFN) signaling pathways in hRSV, are absent in HMPV. That rationalizes the difference in the innate immune response between the two viruses’ infections [14,15,16].

The HMPV genome is made up of negative-sense single-stranded RNA and contains eight genes that code for nine different proteins. From the 3′ to the 5′ end, the genomic gene sequence is N, P, M, F, M2, SH, G, L. The proteins that these genes encode include the N protein: A nucleoprotein. P protein: A phosphoprotein. Matrix protein is the M protein. M2-2 protein: RNA synthesis regulator; M2-1 protein: hypothetical transcription factor; and F protein: fusion glycoprotein. SH protein is a small glycoprotein that is hydrophobic. Adhesion glycoprotein is known as a G protein. L protein: Viral polymerase [17,18].

**Figure 1:**
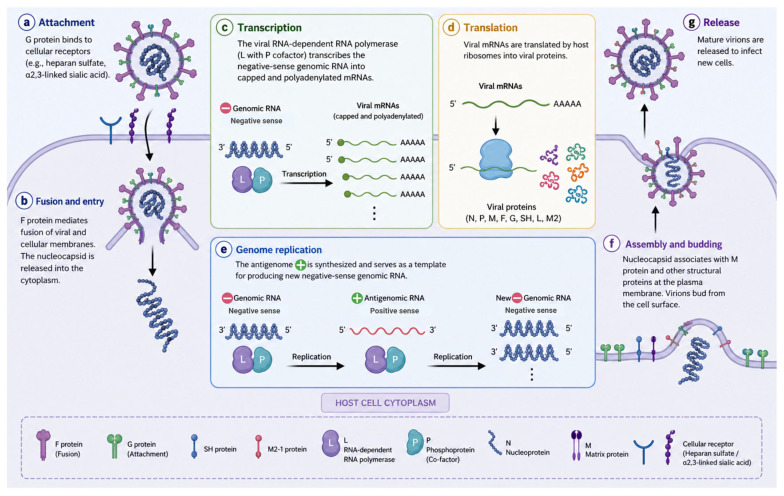
Replication cycle of human metapneumovirus (HMPV). The HMPV replication cycle involves a series of coordinated steps within the host cell cytoplasm: (**a**) Attachment—the viral G protein mediates binding to host cell receptors (e.g., heparan sulfate). (**b**) Fusion and entry—the F protein facilitates fusion of the viral envelope with the host cell membrane, releasing the nucleocapsid into the cytoplasm. (**c**) Transcription—the viral RNA-dependent RNA polymerase complex (L protein with P cofactor) transcribes the negative-sense genomic RNA into capped and polyadenylated viral messenger RNAs (mRNAs). (**d**) Translation—viral mRNAs are translated by host ribosomes to produce structural and non-structural proteins, including N, P, M, F, G, SH, L, and M2 proteins. (**e**) Genome replication—a full-length positive-sense antigenomic RNA intermediate is synthesized and serves as a template for the generation of new negative-sense genomic RNA. (**f**) Assembly and budding—newly synthesized genomic RNA associates with nucleoproteins and viral structural proteins, assembling at the host cell membrane prior to budding. (**g**) Release—mature virions are released from the host cell to infect neighboring cells. Created in BioRender. Hetta, H. (2026) https://BioRender.com/2e2vpcc.

The internal RNA core is surrounded by the M protein and enveloped by a lipid membrane that contains three surface spike glycoproteins (F, G, and SH) with a length of 13–17 nm. A nucleocapsid with a diameter of 17 nm is composed of the interaction of fundamental nucleic acids with other proteins named P, N, L, M2-1, and M2-2 proteins. The viral replication is indicated in Figure 1. Following fusion, the viral nucleocapsid is released into the host cell cytoplasm, where the RNA-dependent RNA polymerase initiates transcription of the negative-sense viral genome into capped and polyadenylated messenger RNAs (mRNAs). These viral mRNAs are subsequently translated by host ribosomes into structural and non-structural proteins required for viral replication and assembly.

In parallel, genome replication occurs through the synthesis of a full-length positive-sense antigenomic RNA intermediate, which serves as a template for the production of new negative-sense genomic RNA. Newly synthesized genomes are encapsidated by nucleoproteins and assembled with viral structural proteins prior to budding from the host cell membrane [19,20,21].

Several proteins are involved in the assembly step of the viral ribonucleoprotein (RNP) complex during viral replication; the first protein is the P protein, which serves as a cofactor that is responsible for the stabilization of the L protein during this step. The second protein is the M protein, which is essential for the assembly and budding of the virus through its interaction with the RNP complex. Also, other proteins are essential for the replication process, such as the N protein, which encapsulates the viral DNA, so it protects it from nuclease activity. Additionally, the M2-2 protein regulates viral transcription and replication and enhances virulence by diminishing host innate protection [22,23,24].

HMPV also possesses mechanisms that contribute to modulation of host immune responses; however, detailed immune evasion strategies are discussed in Section 6 (Immunopathogenesis and Immune Evasion), where they are described comprehensively.

Comparative studies indicate that individuals of distinct genotypes exhibit reduced amino acid and nucleotide similarity (nucleotide 84–86%, amino acid 94–97%) compared to those within the same subgroup (A1 and A2 or B1 and B2) based on F gene sequences [25]. Among the subgroups (A1, A2, B1, and B2), the N gene has the most conservation at both nucleotide (91.2%) and amino acid levels (98.4%), whereas the G gene shows the lowest conservation (79% nucleotide identity and 59.2% amino acid identity) [26].

## 4. Epidemiology

HMPV has been identified on every continent and displays a seasonal pattern. Outbreaks predominantly transpire throughout the spring and winter months, notably from January to March and from June to July in the northern and southern hemispheres, respectively [7,27,28]. One study indicates that the peak of its seasonal cases occurs between March and April, particularly after the RSV and influenza infection seasons [29,30]. Furthermore, its infection season coincides with that of its relative virus, the RSV [31,32,33].

**Figure 2:**
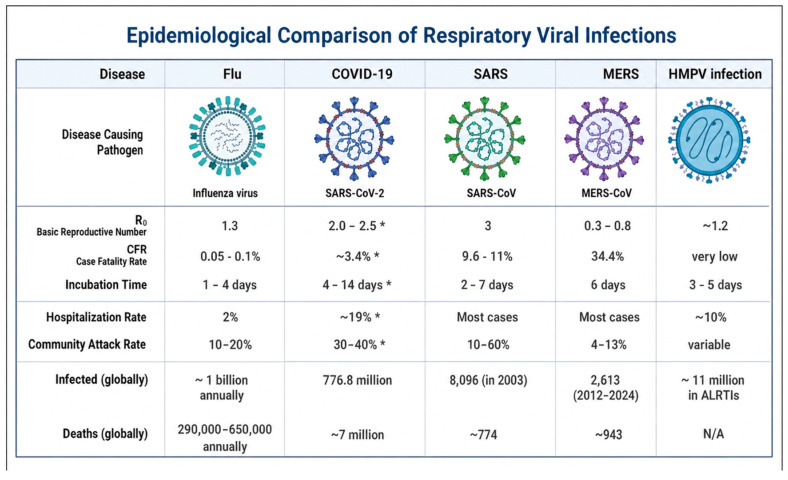
Epidemiological features of HMPV infection among other respiratory viral infections. Comparison of epidemiological metrics like basic reproductive number, case fatality rate, hospitalization and community attack rates, etc., among different viral infections such as influenza viral infection, COVID-19, SARS, MERS, and HMPV. The R_0_ values presented are approximate and derived from limited epidemiological observations; they may vary depending on population, geographic region, and methodological approach. * COVID-19 values reflect early pandemic estimates based on the ancestral SARS-CoV-2 strain; all parameters are time-, context-, and strain-dependent. Created in BioRender. Hetta, H. (2026) https://BioRender.com/2e2vpcc.

HMPV is spread by infectious airborne droplets [34]. The epidemiological pattern among other respiratory viruses is indicated in Figure 2 [35,36,37,38,39,40,41,42]. Seroprevalence studies observe that a significant percentage of children (about 90–100%) are infected at the age of 5–10 years. Also, HMPV reinfection may occur in adulthood [6,12,43]. That is attributed to inadequate immune response during the first infection or reinfection by other genotypes. The virus has an incubation period ranging from 3 to 5 days. Also, it showed maximum virus titers between days 4 and 5 in BALB/c mice and cotton rats [7,44].

Human metapneumovirus (HMPV) is primarily observed in the pediatric demographic, with elevated susceptibility rates in children under the age of 2. In adults, HMPV infection generally manifests as mild influenza-like symptoms; nevertheless, severe consequences, including chronic obstructive pulmonary disease (COPD), may arise, especially in elderly individuals [45]. Adults are more prone to report dyspnea than children [46]. Moreover, HMPV infections have been observed in many immunocompromised individuals, including lung transplant recipients and those with hematological malignancies. Notably, aged-mouse reinfection models demonstrate exaggerated lung pathology driven by impaired CD8^+^ memory T-cell responses, providing mechanistic support for increased disease severity in older adults [47,48,49].

Both A and B genotypes of the virus co-circulate during standard respiratory virus infection seasons [13,50,51]. Recurrent infections with various HMPV genotypes are prevalent [9,52]. Risk factors linked to severe HMPV infection encompass premature delivery, infancy, concurrent nosocomial infection, and other underlying chronic pulmonary, cardiac, or neurological conditions [53]. Research examining the correlation between genotype and disease severity in children has not identified significant associations; yet, there are contrasting results, as certain reports indicate that genotype A may exhibit greater virulence than genotype B, while others indicate the opposite [54,55].

Children infected with HMPV are more prone to necessitate supplementary oxygen, experience extended durations in the intensive care unit (ICU), and receive chest radiography in comparison to HMPV-negative children. About 40% of hospitalized children with HMPV infection possess preexisting high-risk comorbidities, including asthma or chronic lung illness [56]. The average yearly hospitalization rate for HMPV is approximately thrice greater in children under 6 months (3/1000) than in those aged 6 months to 5 years (1/1000). Nosocomial infections have been identified as a mechanism of transmission [57,58].

The yearly hospitalization rate attributable to HMPV infection is analogous to that of influenza and parainfluenza types 1, 2, and 3 collectively [56]. An investigation of one HMPV outbreak in two skilled care institutions indicated an 11% fatality rate linked to severe cases [59]. The seriousness of the illness induced by this newly identified virus underscores the need to comprehend HMPV pathophysiology and accentuates the necessity for vaccine development.

HMPV coinfection is prevalent with many other respiratory viruses such as its relative, the RSV and others, including parainfluenza virus, influenza A and B viruses, coronaviruses, rhinovirus, bocavirus, and enterovirus [27,60,61,62,63,64]. Coinfection has been reported during severe acute respiratory syndrome outbreaks [65], and with bacterial pathogens including *Streptococcus pneumoniae*, *Mycoplasma pneumoniae*, and *Chlamydia pneumoniae* [62]. The relationship between HMPV and other etiological agents is ambiguous; co-infection does not seem to substantially influence the severity of HMPV disease [28,66]. Conflicting data exists concerning the relationship between respiratory syncytial virus-HMPV co-infection and disease severity; certain research suggests heightened ICU admission rates and prolonged hospitalizations due to co-infection, whereas others claim no significant correlation [67,68,69,70,71].

In China, detection rates ranged from 2 to 8% in hospitalized patients during the period 2018–2021, another study in Beijing province compared the variability of virus prevalence during and before COVID-19 pandemic and reported decreasing the percentage from 7.9% at 2018–2019 to 1.7% at 2020–2021 and reported that this decline may be due to strict public health measures during the pandemic which decreased HMPV transmission among hospitalized children, and also because majority of susceptible population are preschool children under five years not greatly affected by school reopening polices during the pandemic. Also, Coinfection with other respiratory viruses was observed, such as RSV and human parainfluenza virus (HPIV). About 37.1 percent of HMPV-positive samples in Beijing province were coinfected with other viruses. Phylogenetic analysis showed genotype predominance shift from B1 to A2b subtype over the last few years.

Pooled molecular prevalence across different studies in China indicated that while overall rates are relatively low (averaging around 4.70%), significant regional variations exist along with notable seasonal peaks primarily during spring months.

In Europe, there was no corresponding rise in the incidence, despite increased HMPV testing during the pandemic [10,72,73].

In New South Wales, Australia, outbreaks were reported during 2018, predominantly in aged care facilities, where logistical challenges complicate outbreak management due to unrestricted movement among staff and residents. The reliance on multiple private pathology laboratories for respiratory screenings has led to inconsistencies in testing for HMPV unless specifically requested by healthcare providers. Clinical manifestations among adults and the elderly often overlap with those of other respiratory viruses; however, symptoms such as wheezing and shortness of breath are particularly common in HMPV infections. The study emphasizes the importance of improving reporting mechanisms for additional respiratory symptoms during outbreaks to enhance understanding of HMPV’s impact on elderly populations. Despite ongoing research into vaccines for HMPV, progress has been limited since its discovery. A combined mRNA vaccine candidate targeting both HMPV and parainfluenza virus type 3 shows promise but faced delays due to the COVID-19 pandemic’s impact on vaccine development efforts [74].

In rural Nepal, research provides critical insights into the molecular characteristics and epidemiological patterns of HMPV infections among symptomatic infants. Multiple HMPV genotypes circulate simultaneously, with an alternating predominant subtype observed over multiple seasons. The study found that HMPV infections predominantly occur from September through March, consistent with findings from other Asian countries. Interestingly, while RSV typically precedes HMPV circulation, the relationship between genotype and virulence remains unclear [75].

In Japan, significant insights into HMPV’s epidemiology have emerged through investigations revealing its potential severity in causing pneumonia cases. A recent case report documented lobar pneumonia caused by HMPV—marking the first documented instance of such a severe manifestation—challenging previous notions that it primarily causes bronchopneumonia. Radiological findings associated with pneumonia due to HMPV include bronchial wall thickening and ground-glass opacities. Genetic analysis has shown the concurrent circulation of various subtypes within populations; the A2c genotype has been identified as prevalent in recent outbreaks in Japan. Co-infections involving other respiratory viruses complicate clinical presentations and may increase illness severity among affected individuals [76,77].

Research on HMPV in India highlights its role as a significant cause of hospitalizations among young children during winter months, with prevalence rates ranging from 4 to 12% across various regions. Interestingly, while earlier research suggested that younger children are primarily affected by HMPV, recent findings reported a mean age of 24.6 years among patients experiencing severe disease related to HMPV infections. Genetic analysis reveals the circulation of multiple subtypes in India, including A2b and B2; notably, A2b was found to be the most prevalent at 82.1% during certain periods studied. The overall burden of HMPV in the general population remains unclear due to limited studies; however, it is increasingly recognized as an important etiological agent responsible for lower respiratory tract infections (LRTIs) in children [78,79].

Also in Vietnam, HMPV’ studying offers valuable insights that can significantly impact public health and patient care. Over the years, researchers have observed interesting shifts in the circulating genotypes of HMPV. For instance, genotype A2b was the dominant strain before 2009, while A2c took over from 2011 onwards. This change suggests that HMPV is not static; it evolves and adapts, which is why ongoing monitoring of its genetic diversity in Vietnam is so important. Understanding these patterns can help us grasp how the virus behaves differently in various regions and inform our response strategies. When it comes to clinical manifestations, HMPV infections often look a lot like those caused by respiratory syncytial virus (RSV) or other viral infections. Around 52% of patients with HMPV experience wheezing, a finding that aligns with previous research. This overlap highlights the need for better diagnostic tools to distinguish between these infections, as accurate identification can lead to more effective treatment. The study also sheds light on the broader epidemiology of HMPV. The simultaneous circulation of multiple genotypes can influence how frequently and severely people get sick. This underscores the necessity for targeted public health interventions and possibly even vaccination strategies tailored to these evolving strains. Interestingly, researchers found that the F gene of HMPV shows a relatively stable evolutionary rate. This stability might play a role in how the virus spreads. However, there are differences between the F and G genes that suggest they interact with our immune systems in distinct ways. Understanding these interactions could be crucial for developing effective vaccines in the future [80].

In addition to the regions discussed above, HMPV has been reported globally across diverse geographic settings. Studies from North America and Europe have demonstrated consistent seasonal circulation, typically peaking in late winter and early spring, with patterns overlapping those of RSV and influenza. In Africa and the Middle East, limited but emerging data suggest similar epidemiological trends, although underreporting and limited surveillance infrastructure may obscure the true burden of disease. These findings highlight the global distribution of HMPV and emphasize the need for more comprehensive surveillance across underrepresented regions. These geographically diverse findings highlight the widespread circulation of HMPV worldwide (Table 1).

The transmissibility of HMPV remains incompletely characterized, and estimates of its basic reproduction number (R_0_) are limited and highly context-dependent. Available data are largely derived from observational epidemiological studies rather than robust mathematical modeling [35,82], which introduces uncertainty in direct comparisons with other respiratory viruses. Unlike influenza and SARS-CoV-2, which often exhibit seasonal dominance of a single strain, HMPV is characterized by the co-circulation of multiple genetic lineages. This pattern may reflect partial cross-protective immunity, regional heterogeneity in viral dynamics, and distinct evolutionary pressures rather than low transmissibility per se. Consequently, caution should be exercised when interpreting R_0_ estimates and comparing transmissibility across respiratory viruses.

## 5. Clinical Characteristics

The clinical signs of HMPV infection sometimes resemble those of the infection by its relative, the respiratory syncytial virus, especially in young infants. Patients with HMPV are generally diagnosed with bronchiolitis, bronchitis, and pneumonia, presenting with usual symptoms such as Pyrexia, Coughing, Hypoxia, Upper respiratory tract infection, infection of the lower respiratory tract, and Stridor [83,84].

Fever lasts approximately 10 days on average for people with HMPV, peaking throughout the illness [85]. When a young person is re-infected, the symptoms are usually mild and similar to a cold or influenza, with fever appearing in a small percentage of cases. However, severe symptoms like pneumonitis, which can be fatal, can be seen in older adults [46,86].

In children with wheezing, HMPV is found in about 8% of cases and is associated with otitis media in 50% of cases [87,88]. Numerous studies on children with lower respiratory tract infections linked to HMPV have identified wheezing as a common clinical symptom [89]. Additionally, HMPV infections have been shown to exacerbate chronic obstructive pulmonary disease (COPD) and can exacerbate asthma in both adults and young children [47]. People with COPD are more susceptible to contracting HMPV [90,91,92]. Few reports indicate that HMPV infection in children may be associated with various CNS disorders, including febrile seizures and severe encephalitis [93].

Although asymptomatic children have been found to have HMPV by RT-PCR, their virus levels were significantly lower than those of sick children [94]. Regardless of the viral genotype, elevated HMPV viral loads are linked to worsening illness and faster disease progression [95]. After acute illness begins, elevated virus shedding may persist for one to two weeks [96,97].

In at-risk groups, severe cases of pneumonia linked to HMPV have been documented. For instance, a child undergoing chemotherapy for acute lymphoblastic leukemia developed fatal pneumonia associated with HMPV [52]. In a different example, a recipient of an allogeneic hematopoietic stem cell transplant experienced severe alveolar cell damage due to interstitial and intra-alveolar pneumonitis brought on by HMPV infection [48]. Significantly higher rates of morbidity and death are associated with infection within the first week after transplantation [97].

HMPV can cause a variety of illnesses in lung transplant recipients, ranging from mild upper respiratory infections to serious lower respiratory infections [98,99]. A prospective study of individuals with significant physical and cognitive impairments found that modest to moderate increases in C-reactive protein (CRP) levels, decreased peripheral blood lymphocytes, and an elevated monocyte ratio were indicative of the early stages of HMPV infection. When the sickness subsided, immunological indicators returned to normal, but elevated CRP levels remained elevated for a long time [85]. Some HMPV-infected hospitalized children have also shown leukopenia and leukocytosis [100].

## 6. Immunopathogenesis and Immune Evasion

Chronic infection by human metapneumovirus (HMPV) may result from a subdued and protracted immunological response, coupled with diminished cytotoxic T-lymphocyte function that obstructs viral elimination after initial infection [101]. HMPV disrupts superantigen-mediated T cell activation by infecting dendritic cells, hence limiting the proliferation of antigen-specific CD4+ T cells and hindering the development of long-term immunity [102].

Human metapneumovirus is a less potent inducer of a number of cytokines, such as interleukin (IL)-12, tumor necrosis factor alpha (TNF-α), IL-6, IL-1β, IL-8, and IL-10, than influenza and the respiratory syncytial virus [103,104]. In animal models such as BALB/c mice and cotton rats, HMPV infection causes changes in pulmonary inflammation that lead to increased levels of interleukins (IL-2, IL-8, IL-4), interferon (IFN-α), macrophage inflammatory protein 1α, and monocyte chemotactic proteins in lung tissue and bronchoalveolar lavage fluid. In the perivascular and peribronchiolar areas, these changes result in inflammation and infiltration [47,105].

Histopathological analyses reveal the presence of smudge cells, intra-alveolar foamy macrophages, hemosiderin-loaded macrophages, alveolar damage, and hyaline membrane disease associated with HMPV infection [98]. The specific role of toll-like receptor-mediated signaling in the host’s defense against pulmonary HMPV infection is unclear, despite the fact that HMPV infection triggers toll-like receptor-dependent cellular activation. According to another study, following intranasal infection with HMPV, MyD88-deficient mice showed significantly less pulmonary inflammation and associated disease than wild-type C57BL/6 mice [106].

However, there is currently no conclusive evidence that HMPV is limited to the respiratory tract during infection or that it can cause systemic illnesses. One study found HMPV in middle ear fluid, and another isolated HMPV RNA in the brain tissue of a patient who died of encephalitis, suggesting possible systemic involvement. Further investigation is needed to clarify these results [87,107].

**Figure 3:**
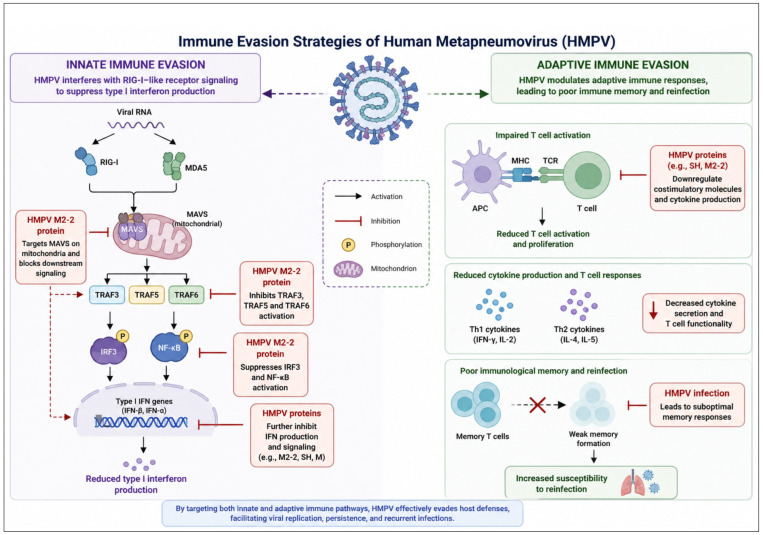
Mechanisms of innate and adaptive immune evasion by human metapneumovirus (HMPV). HMPV evades host immune responses through coordinated interference with both innate and adaptive immunity. In the innate immune pathway, viral RNA is recognized by pattern recognition receptors RIG-I and MDA5, which signal through mitochondrial antiviral signaling protein (MAVS). The viral M2-2 protein targets MAVS, disrupting downstream signaling and inhibiting the activation of adaptor molecules TRAF3, TRAF5, and TRAF6. This leads to suppression of key transcription factors, including interferon regulatory factor 3 (IRF3) and nuclear factor kappa B (NF-κB), resulting in reduced production of type I interferons (IFN-α/β). Additional viral proteins, such as SH and M proteins, further contribute to inhibition of interferon signaling. In the adaptive immune response, HMPV impairs antigen presentation and T-cell activation by modulating host immune signaling pathways. This results in reduced T-cell proliferation, decreased cytokine production, and impaired differentiation of effector T cells. Consequently, the formation of long-lasting immunological memory is compromised, leading to increased susceptibility to reinfection. Created in BioRender. Hetta, H. (2026) https://BioRender.com/2e2vpcc.

As seen in Figure 3, the human metapneumovirus (HMPV) uses a variety of strategies to avoid the host immune response, mostly by using structural proteins such as the G and SH glycoproteins. The G protein has demonstrated the ability to suppress type I interferon (IFN-I) responses in vitro and in vivo, and it is crucial for the initial engagement with host cells. Variations in many molecules, including CCL3, CCL4, VEGF, TNF, IL-17, and CXCL2, show that this suppression is associated with changes in neutrophil recruitment to the alveolar space. The G protein encourages the migration of polymorphonuclear cells that release cytokines like IL-13 and IL-5 by activating the thymic stromal lymphopoietin (TSLP) pathway.

Research on human monocyte-derived dendritic cells (MDDCs) showed that HMPV infection led to lower maturation levels and a reduced ability to present HMPV antigens to naïve T cells, in contrast to MDDCs infected with a mutant HMPV that lacked both SH and G proteins. This implies that these proteins prevent T-cell activation. While the G protein has little ability to replicate in monocyte-derived dendritic cells (MDDCs), it facilitates viral replication in airway epithelial cells.

Studies using HMPV mutants in vivo show that the G protein’s deletion increases the production of type I IFN and boosts immunological responses. Because it inhibits STAT1 phosphorylation, which is essential for subsequent antiviral signaling, and adversely modulates the NF-kB pathway. Following HMPV infection, this inhibition leads to a decreased inflammatory response. Also, the SH protein plays a critical role in immune evasion through targeting this pathway.

M2-2 and other supplemental proteins block mitochondrial antiviral signaling (MAVS), which is necessary for the activation of the NF-kB and IRF3 pathways, hence facilitating immune evasion. Its inhibitory function in innate immune responses is demonstrated by the increased cytokine and interferon production that occurs when M2-2 is absent.

The effects of HMPV on the innate arm of immunity have been studied in a number of immune cells, such as alveolar epithelial cells and macrophages. HMPV infection of alveolar epithelial cells causes severe airway remodeling and damage. These cells respond to infection by using pattern recognition receptors (PRRs) like RIG-I and MDA-5 to recognize HMPV. These receptors start inflammatory signaling pathways and the release of cytokines. Type I IFN signaling can be blocked by HMPV on various levels, affecting vital proteins associated with this system.

Other than immunity, HMPV has been affecting cellular metabolism. It was reported that HMPV infection downregulates vital metabolic enzymes and disrupts tricarboxylic acid cycles, so it interferes with viral replication. Also, it showed reduced production of antioxidant enzymes and increased oxidative stress markers within infected cells. It also led to increased alveolar epithelial cells’ apoptosis during the first week post-infection; some infected cells evaded apoptosis, hence promoting viral persistence. This persistence has been demonstrated in mouse models and particular immunocompromised patients.

Another cell that was studied upon HMPV infection is the alveolar macrophage, which showed an essential role in the early stage of infection, as its pre-inoculation depletion enhanced lung viral replication and pulmonary inflammation, in contrast to post-inoculation depletion that showed no effect on lung viral replication [108,109].

## 7. Diagnostics

**Figure 4:**
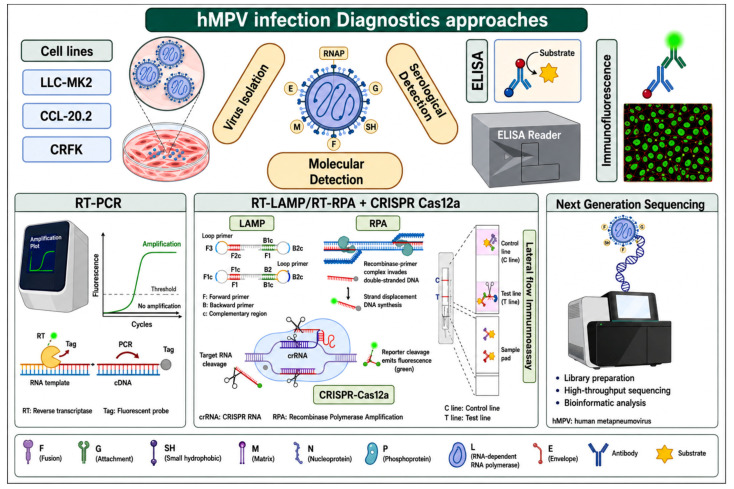
Current and under-research diagnostic approaches for HMPV infection. Several approaches are in use or under investigation to effectively diagnose HMPV viral infections. the 1st approach is the viral isolation using some cell lines (LLC-MK2, CRFK), the 2nd approach is the serological detection using techniques such as ELISA and Immunofluorescence, and the 3rd approach is the molecular diagnosis using either traditional thermal cycling-based amplification (RT-PCR, multiplex RT-PCR, RT-qPCR) or Isothermal amplification (LAMP, RPA) that also can be combined with CRISPR-Cas 12a system as a detection system and results can be seen visually by lateral flow immunoassay methodology. Furthermore, metagenomic sequencing may be used to unravel the complete viral genomes and can identify coinfection with other viruses. Created in BioRender. Hetta, H. (2026) https://BioRender.com/2e2vpcc.

Early diagnosis is essential for HMPV viral infection management through developing effective measures to control outbreaks and facilitate early patient care. HMPV is one of the viruses that are commonly neglected, and its diagnosis is not included in the routine of many hospitals [110]. Several diagnostic approaches, as shown in Figure 4, will be presented here, whether they are currently in use; the diagnostic as well as other facets of research have shown significant results.

### 7.1. Virus Isolation

HMPV, similar to other viruses, needs cell lines to be isolated, so several cell lines have been used in its cultivation and extraction, for example, Vero cells, Hep G2 cells, 293 cells, and LLC-MK2 cells [12,45,111,112]. Additionally, recent investigations showed that other cell lines, such as CCL-20.2 (human Chang Conjunctiva cell line [clone 1-5C4]) and CRFK cell line (feline kidney cell line), are the optimal cell lines for HMPV proliferation [113]. In cell culture, HMPV has a slow growth rate that is marked by delayed cytopathic effects like cellular rounding, cellular dissociation from the culture matrix, and the formation of small syncytia. It showed lower sensitivity and specificity compared to RT-PCR. Thus, this methodology is often used in research, and it is seldom utilized for its diagnosis [72].

### 7.2. Serological Diagnostics

Serological diagnostics of HMPV include the identification of the virus antigen with anti-HMPV antibodies in direct fluorescence or ELISA tests, which is commonly used in conjunction with cell culture techniques [111]. Although there is a high similarity between amino acid sequences of the F protein common to HMPV and its relative, the respiratory syncytial virus has led to the creation of restricted serological techniques for identifying HMPV-specific antibodies [72].

### 7.3. Molecular Diagnostics

The second methodology, and currently the standard technique used for viral detection, is RT-PCR. As real-time RT-PCR (rRT-PCR), compared to cell culture detection-based methodology, reported sensitivity and specificity of 68% and 99%, respectively [114]. This technique depends on Genomic areas with significant sequence homology, like the F and N genes, as molecular markers in HMPV detection. Using a detection limit of 1000 copies per reaction, Li et al. created a multiplex RT-PCR technique (mRT-PCR) that can identify sixteen pathogens, including HMPV, associated with acute respiratory tract infections [115,116]. mRT-PCR multiplex with sensitivity and specificity rates of 100% and 96%, respectively, has emerged as a more effective and sensitive method for HMPV detection than real-time RT-PCR and can simultaneously detect HMPV and other pathogens associated with acute respiratory tract infections [117].

mRT-PCR’s ability to detect co-infections, especially those with low viral loads that could go undetected by cell culture or immunostaining techniques, is a crucial advantage [118]. Many clinical labs are currently unable to perform routine diagnostic RT-PCR for HMPV identification. In order to diagnose HMPV infections quickly and accurately, a combination of direct fluorescent antibody methods and immunofluorescence tests should be used as the first diagnostic method. If the results are negative, RT-PCR should be performed on the negative specimens [119]. Another technique is RT-qPCR, which showed higher sensitivity and lower contamination risk compared to RT-PCR, so it has become the gold standard diagnostic technology.

In 2008, Lu et al. developed a TaqMan-based RT-qPCR method that attained a detection limit of 10 copies/μL, identifying 19.62% of clinical samples as positive for HMPV, compared to 13.92% with traditional RT-PCR. Furthermore, digital microfluidic technology has been integrated into RT-qPCR platforms for pathogen detection, achieving sensitivities of ≤150 copies/reaction off-chip and ≤120 copies/reaction on-chip [72]. Additionally, with the appearance of Isothermal amplification techniques such as Loop-mediated isothermal amplification, which are characterized by their exceptional sensitivity and ease of use. Song et al. developed LAMP primers aimed at the M genes to distinguish between HMPV genotypes A and B, attaining detection limits of 4.33 copies/μL and 3.53 copies/μL, respectively, thereby demonstrating markedly improved sensitivity relative to traditional RT-PCR techniques. Wang et al. developed a technique utilizing primers specific to the N gene, with sensitivity levels under 10 copies/μL. LAMP functions at a consistent temperature (~65 °C) without the need for intricate thermal cycling apparatus and exhibits enhanced specificity through the use of multiple primer pairs [72].

Furthermore, recombinase-aided amplification (RAA) has emerged as a novel technique noted for its user-friendliness and enhanced amplification efficiency; Jiao et al. devised primers targeting the HMPV N gene for an RAA method with a detection limit of 100 copies/μL—surpassing the sensitivity of commercial RT-qPCR methods—and necessitating a reduced runtime of 15 min at 39 °C [72]. Moreover, CRISPR-Cas12a technology has attracted interest in nucleic acid detection owing to its resilience; it can be integrated with isothermal amplification methods like LAMP or RAA to improve sensitivity. Qian et al. developed a technique for detecting HMPV RNA utilizing RT-RPA in conjunction with CRISPR-Cas12a and lateral flow assays, attaining a detection limit of under 700 copies/mL in 30 min [72].

Additionally, metagenomic next-generation sequencing (mNGS) has evolved as a high-throughput diagnostic approach adept at identifying novel viruses when conventional methods are inadequate; it can accurately amplify whole viral genomes but necessitates reverse transcription into cDNA before sequencing. Xu et al. employed nanopore metagenomic sequencing to examine nosocomial transmission of HMPV in hematological patients [72,120].

## 8. Current and Potential Management Methods

**Figure 5:**
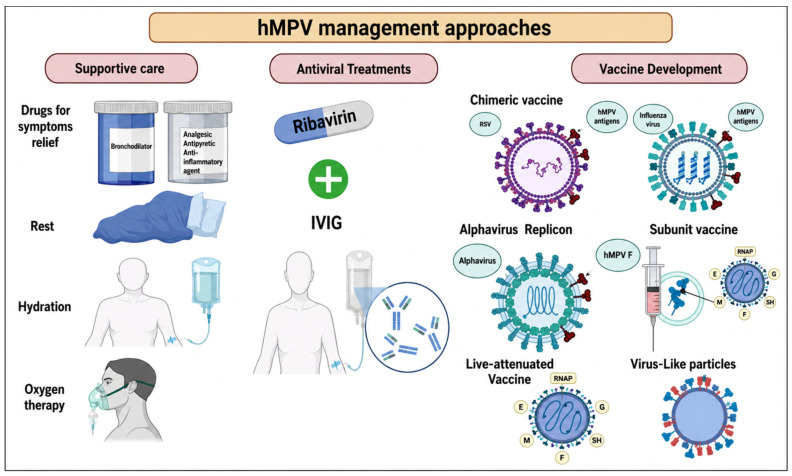
Current and under-research approaches for HMPV infection. HMPV infection management primarily focuses on symptom relief using different drugs (Ibuprofen, Acetaminophen), hydration, and, in some cases, oxygen support. Another approach being investigated is using antiviral Ribavirin with IV immunoglobulin. The last approach is vaccine development, in which several types of vaccines are being investigated, like chimeric vaccines (RSV, Influenza virus, Alphavirus expressing HMPV antigens), subunit vaccines using HMPV F protein, Live-attenuated HMPV vaccines, and lastly VLP expressing HMPV proteins. Created in BioRender. Hetta, H. (2026) https://BioRender.com/2e2vpcc.

The existing treatments for human metapneumovirus (HMPV) infection, as indicated in Figure 5, are predominantly supportive in nature [121]. Numerous papers have investigated the possible application of ribavirin, immunoglobulin, fusion inhibitors, and small interfering ribonucleic acids for the treatment and management of HMPV infection [122,123,124,125,126,127,128,129]. Also, a recent study showed that probenecid significantly inhibited HMPV replication in vitro, and probenecid prophylaxis or treatment reduced HMPV replication in BALB/c mice [130].

Several vaccination options for HMPV have been evaluated in rodent and non-human primate models. Despite the candidates demonstrating encouraging outcomes, none have been evaluated in human subjects to date. Concerns have emerged regarding a heat-inactivated viral vaccination for HMPV, which was observed to exacerbate pulmonary illness in murine models [131]. T-cell epitope vaccines have shown efficacy in mitigating immunomodulation induced by HMPV exposure. For example, murine subjects vaccinated with an HMPV cytotoxic T cell epitope vaccine exhibited reduced levels of Th1 and Th2 cytokines relative to non-immunized mice after the HMPV challenge [132].

Chimeric vaccines for HMPV infection have been assessed in research investigations. These vaccines were studied in hamsters and African green monkeys and demonstrated induction of neutralizing antibodies and protected against wild-type infections [133,134]. Moreover, subunit vaccines employing the fusion protein of HMPV have been successful in eliciting cross-protective immunity against HMPV challenges in hamsters [135]. Multiple HMPV F subunit vaccines have demonstrated robust protective efficacy in trials involving rats, hamsters, and non-human primates [136,137,138].

An investigation has examined HMPV virus-like particles (VLPs) that replicate the characteristics of the viral surface for potential application as a vaccine candidate. In murine models, these VLPs effectively elicited a robust humoral immune response against both heterologous and homologous pathogens [139]. Although the HMPV-VLP vaccine shows potential, additional research is necessary to create a vaccine that is effective against all HMPV subgroups.

The advent of plasmid-based reverse genetics techniques has markedly enhanced initiatives to create a live vaccination for HMPV infection. Recombinant HMPVs with deletions in the SH, G, or M2-2 genes have been assessed for viral replication levels, indicating that these deletions do not influence the virus’s immunogenicity or antigenicity [22,140]. One study created a live attenuated vaccine strain of HMPV by modifying the glycosylation location of the F protein; this vaccine conferred complete protection against homologous virus challenges and partial protection against heterologous challenges even at 56 days post-inoculation [141]. Importantly, recent evidence shows these deletions produce strain-dependent phenotypes, where an SH-deleted virus derived from the A1/C-85473 backbone demonstrated strong attenuation, robust immunogenicity, and cross-protective efficacy, underscoring the need to consider viral genetic background in LAV design [142].

Another study reported a novel chimeric influenza vaccine expressing a partial HMPV fusion protein. This vaccine, termed RFLU-HMPV/F-NS generated using reverse genetics and showed a robust immune response in mice. It provides protection against both wild-type HMPV and Influenza viruses, indicating it as a probable candidate for HMPV vaccination [143]. Other research studies have produced chimeric versions of recombinant HMPV by replacing specific proteins (nucleoprotein or phosphoprotein) with those from avian metapneumovirus (AMPV). These chimeras exhibited improved replication in vitro while maintaining attenuation in vivo, making them promising candidates for further clinical evaluation [134,144].

Another probable vaccine candidate is the bivalent live attenuated vaccine combining HMPV and respiratory syncytial virus (RSV) proteins.

This type has shown protective efficacy in mice. Dependance on the Metavac^®^ platform, this vaccine was created. This vaccine induced strong neutralizing antibody responses against both viruses and reduced lung inflammation upon infection [145].

Other studies emphasized the importance of the prefusion conformation of the HMPV fusion protein in eliciting potent neutralizing antibodies. Dependance on that Researchers designed Vaccines to stabilize this conformation. This type of vaccine demonstrated effective immune responses and protection against HMPV infection in animal models [146]. Another innovative approach that used alphavirus replicons to develop HMPV vaccines. These replicons have shown immunogenicity and protective efficacy in preclinical studies, suggesting a viable strategy for future vaccine development [147].

Another example of chimeric vaccines is the chimeric fusion proteins that are generated by incorporating neutralizing epitopes from both HMPV and human respiratory syncytial virus (hRSV). The study reported it as a potential immunogen to induce cross-protective immunity against these closely related viruses. Either Postfusion-stabilized HMPV F-bearing antigenic site II of hRSV or Prefusion-stabilized hRSV F bearing antigenic site IV of HMPV induces antibodies that cross-neutralize and protect against both infections [148]. These findings highlight the potential for effective vaccination strategies against HMPV, which remains a significant cause of respiratory infections, particularly in young children and immunocompromised individuals. Further studies and clinical trials will be essential to validate these candidates’ safety and efficacy.

## 9. Conclusions

In conclusion, human metapneumovirus (HMPV) is an important but frequently neglected respiratory pathogen that has considerable clinical significance for individuals of all ages. The findings highlight the necessity for increased awareness among healthcare professionals about the potential severity of HMPV in both children and adults. Recent findings indicate that HMPV has the potential to cause significant respiratory illness in immunocompetent individuals, highlighting the need for additional investigation into its epidemiology and pathophysiology. With the ongoing global circulation of HMPV, especially during peak seasons, there is a pressing need for improved surveillance, precise diagnostic methods, and robust public health strategies to lessen its effects on at-risk groups. Future studies should concentrate on elucidating the genetic diversity of HMPV and its correlation with disease severity to guide vaccine development initiatives and enhance patient outcomes.

## Figures and Tables

**Table 1:** Epidemiological overview of HMPV across different regions.

Region/Country	Study Period	Key Findings	Reference
China	2018–2021	Detection rates 2–8%; decline during COVID-19	[10,72,73,81]
Australia (NSW)	2018	Outbreaks in aged care facilities; seasonal peak late winter	[74]
Nepal	Multi-season study	Co-circulation of genotypes; peak Sept–March	[75]
Japan	2017–2022	Emergence of A2c genotype; severe pneumonia cases reported	[76,77]
India	Recent years	Prevalence 4–12%; A2b predominant	[78,79]
Vietnam	2007–2017	Genotype shift (A2b → A2c); ~52% wheezing cases	[80]
North America	Various	Seasonal peak late winter–spring; significant pediatric burden	[32,56]
Europe	Recent studies	Stable incidence despite increased testing	[10,72,73]

## Data Availability

No new data were created or analyzed in this study. Data sharing is not applicable to this article.
